# Seroprevalence of anti-HBc, risk factors of occupationally acquired HBV infection and HBV vaccination among hospital staff in Poland: a multicenter study

**DOI:** 10.1186/s12889-019-6628-1

**Published:** 2019-03-12

**Authors:** Maria Ganczak, Katarzyna Topczewska, Maria Budnik-Szymoniuk, Marcin Korzeń

**Affiliations:** 1 Department of Epidemiology and Management, Faculty of Medical Sciences, Pomeranian Medical University, Zolnierska 48, 71-210 Szczecin, Poland; 2 Department of Epidemiology and Management, Faculty of Medical Sciences, Pomeranian Medical University, Zolnierska, 48, 71-210 Szczecin, Poland; 3 Department of Social Nursing, Faculty of Medical Sciences, Collegium Medicum, ul. Łukasiewicza 1, 85-821 Bydgoszcz, Poland; 4 Department of Methods of Artificial Intelligence and Applied Mathematics, Faculty of Computer Science and Information Technology, West Pomeranian University of Technology, Zolnierska 46, 71-210 Szczecin, Poland

**Keywords:** Seroprevalence, HBV, Risk factors, Occupational exposure, Hospital staff, Poland

## Abstract

**Background:**

Due to numerous blood exposures hospital staff are at risk of acquiring hepatitis B virus (HBV) infections. This study aimed at estimating prevalence of HBV, associated risk factors and HBV vaccination among Polish health care workers (HCWs).

**Methods:**

A cross-sectional sero-survey was conducted (October 2016–January 2018) in 10 randomly selected hospitals from two provinces: of low and high incidence of HBV, with the use of an anonymous, self- administered questionnaire. Blood samples were screened for hepatitis B core antibodies (anti-HBc) with enzyme immunoassay.

**Results:**

Of the 306 participating HCWs, 88.6% were females, 69.9% nurses (mean age 47.8 ± 9.0 years). HBV vaccination was reported by 94.2%, participants (4.7% with 2 doses, 58.1% with 3 doses, 37.2% took a booster), but of these 75.1% reported no post-immunization serology. The sero-prevalence of anti-HBc was 12.1% (95%CI 8.4–15.7%); only 11.1% had ever screened themselves for HBV infection. Out of 37 anti-HBc positive HCWs, 29 reported being vaccinated for HBV; 10.5% vaccinated HCWs were anti-HBc positive. Regarding other occupational risk factors, 27.8% had experienced a sharp injury (SI) in the last year, 80.0% of incidents were not reported. The use of safety devices (SD) was 86.3%; 35.9% participants used to recap a needle. Older age (OR = 4.24), lack of HBV vaccination (OR = 7.42), working at the province of high HBV incidence in the general population (OR = 2.69) were each predictors of participant’s HBV infection.

**Conclusions:**

High anti-HBc seroprevalence was found in hospital staff with older generation particularly constituting a risk group. Unsatisfactory vaccination coverage and the use of SDs, needle recapping and under-reporting of SIs were main modifiable risk factors regarding HBV infection. The study provides evidence of the protective role of HBV vaccine, as well as the possible effect of HBV incidence in the general population on HCW’s anti-HBc seropositivity. Universal vaccination, followed by strict policies to confirm immunity, better compliance with infection-control practices and widespread implementation of SDs should be enforced to protect hospital staff from occupationally acquired HBV infections.

**Electronic supplementary material:**

The online version of this article (10.1186/s12889-019-6628-1) contains supplementary material, which is available to authorized users.

## Background

Occupationally acquired blood-borne infections (BBIs) caused by hepatitis B virus (HBV) have been described worldwide in the healthcare sector [1–3]. The risk of acquisition of this infection in an unvaccinated individual after a single exposure is estimated 32–67% when blood is positive for both hepatitis B surface antigen (HBsAg) and envelope antigen (HBeAg) and 6% - when HBeAg is negative [3]. The World Health Organization reported that 66,000 HBV infections may have occurred in 2000 among healthcare workers (HCWs) due to their occupational exposures, mainly sharps injuries (SIs) [2]. A four times greater probability of contracting HBV infection has been reported for a HCW as compared to the general population. However, due to the asymptomatic course of viral hepatitis, some infected HCWs may be unaware of their serological status [3] until significant liver damage, such as liver cirrhosis and hepatocellular carcinoma has occurred. Although the number of new cases of occupationally acquired viral hepatitis has fallen in Poland over the last few years, still it was responsible for more than a half of infections diagnosed among HCWs [4]. The majority of cases were reported among hospital staff; almost every second case was detected among nurses.

The risk of an occupationally acquired HBV infection in a HCW depends on the number of blood exposures sustained during medical procedures, the risk of transmission at each exposure and the prevalence of HBV in general population, particularly in hospitalized patients [5]. Personal factors such as older age, long practice duration, high number of exposures, specifically sharps injuries (SIs) experienced by a HCW, lack of training in infection control, and not using protective equipment con-tribute to contracting HBV at hospital setting [1, 3, 6–12]. Specific job categories, such as a nurse or a laboratory technician, were also found to be associated with a higher risk of current HBV infection [7].

Hospital staff are known be exposed to contaminated blood due to numerous SIs, most often caused by needles; the vast majority of these staff experience more than one incident during their professional career [12–16]. In addition to this, underreporting of these risk exposures is found to be widespread [12, 14–18], which represents a missed chance for initiating implementation of prevention strategies, such as hepatitis B immune globulin and HBV vaccine [19]. Above mentioned factors can lead hospital staff to a higher level of risk of contracting a BBI, including HBV, than that of HCWs working in some other types of facilities.

Hepatitis B incidence (acute and chronic cases) has decreased dramatically in Poland in the last 40 years. However, it has been estimated that there are still about 350,000 carriers of HBsAg in the general population, with the broad variation at the level of particular provinces [20]. According to some previous studies, hospitalized patients have shown higher seroprevalence rates of HBV than observed in the general population, with patients from internal medicine [21], as well as surgical [22], and emergency departments [23], reporting the highest rates. The prevalence of HBsAg was 0.6% among a sample of surgical and gynecological patients from Polish hospitals surveyed in 2008–2009 [22]. According to the more recent study, from 2013, the prevalence of total antibodies to the hepatitis B core antigen (anti-HBc total) among Polish primary care clinics patients was 10.3% [24].

In the European Union (EU), concerns about BBIs resulting from SIs and poor compliance with recommended infection control procedures in the health care sector lead to developing specific measures to protect employees where there is an occupational risk of infection from sharps. A EU Directive on the prevention of SIs (Council Directive 2010/32/EU) was adopted into European law in May 2010 [25] and became mandatory three years later [13]. The Directive recommends adherence to a list of measures, including the use of safety engineered devices (SDs), to eliminate risk where it exists to prevent injury, as well as the universal vaccination against HBV [13, 25]. Five years after the implementation of the Directive there is no Polish data available on the use of SDs at the hospital level. Furthermore, despite the fact that a safe, effective and highly acceptable HBV vaccine has been available for over 30 years [3] and has been provided at no cost for Polish HCWs since 1989, a number of hospital staff still remain unvaccinated [26, 27].

Although surveys assessing the risk of occupationally BBIs have been conducted among HCWs in the region, most papers have concentrated on the frequency and circumstances of occupational exposure to blood [17, 28–30]. To the best of our knowledge, there has been no recent multicenter survey, conducted in the Central Europe after the implementation of the Council Directive, which assessed the epidemiology of SIs and the use of SDs in the context of HBV seroprevalence and vaccination of HCWs. Thus, the current study was aimed at investigating the seroprevalence and the possible risk factors of HBV infection among hospital staff from selected hospitals located in two Polish provinces: Kuyavia, in which the incidence of hepatitis B infections is among the highest, in the last 10 years and West Pomerania in which it is among the lowest [20, 31].

## Methods

### Setting

Of 16 Polish provinces, two were selected: one in which the total incidence of hepatitis B infections is among the highest, and one in which it is among the lowest (Kuyavia and West Pomerania with the total incidence in 2005–6.1 and 2.5 per 100,000 respectively and in 2015–15.9 and 9.4 per 100,000 respectively) [31]. A multistage proportional stratified sampling method was used in every province. The sampling frames included lists of hospitals obtained from the local health departments. Only hospitals which comprised of surgical, gynecological, pediatric and internal medicine wards were included. Firstly, in Kuyavia province (a study province with the high incidence of HBV infections) 2 hospitals were randomly selected out of 22 hospitals which fulfilled the inclusion criteria. Then, 8 control hospitals (1:4 ratio of a study/control group) were randomly selected in the West Pomeranian province out of 25 hospitals in the region. As a next step, a random sampling of one ward category (e.g. surgery, gynecology, internal medicine, pediatric ward, etc.) was made for selected hospitals with more than one category of ward. This multistage stratified sampling scheme has been used for our studies published previously [11, 17, 22].

#### Data collection

The study was conducted between October 2016 and January 2018.

#### Study population

consisted of hospital staff: physicians, nurses, midwives, paramedics, and cleaners. It was a census sample of all HCWs present in the wards at the time the study was conducted, except those on leave.

#### Study design

This was a cross-sectional sero-epidemiological multicenter survey.

#### Data collection

Data were collected by an anonymous self-administered questionnaire distributed by the study members. The questionnaire was developed by the authors and has not been previously published elsewhere. It included 23 closed questions measuring the following variables: 1. Socio-demographic data (age, gender, job category, length of practice, number of working hours per week, type of ward, province), 2. Occupational risk factors for acquiring BBI by SIs, i.e. *personal risk factors*: training in infection control, work practices (recapping), immunization against HBV, reporting of SIs; *equipment risk factors*: use of safety engineered devices (SDs) and use of gloves; 3. Exposures to SIs (number of injuries during the preceding 12 months); 4. Non-occupational risk factors: ever having blood transfusion, having surgery, tattooing, having multiple sexual partners; see Additional file 1 for more detailed description. The age of participates was classified into four categories: < 35, 36–45, 46–55, and > 55 years. Length of practice was categorized as ≤10, 10–20, 20–30, and > 30 years. Number of working hours per week was separated into two categories of ≤170, and > 170.

A pilot study among 20 nurses from one selected hospital was undertaken in order to test the feasibility of the questionnaire. Participants’ comments only referred to the small number of questions and were related to the modification of item wording, specific clarifications, changing the order in which questions were presented, adding an open question or altering the instrument format. Therefore, after reviewing the comments and making a number of amendments to improve the clarity of the questions, the results from the pilot study were included in the main survey.

Exposure to SIs in the survey questionnaire was defined as being injured by a needlestick or cut with a sharp object within the last year prior to the survey.

### Blood sampling

The aim of this study was to estimate the prevalence of HBV, associated risk factors and HBV vaccination uptake among Polish HCWs, rather than to assess HBV immunity post-vaccination among HCWs who reported completing the HBV vaccination series. The latter was assessed in our previous study [32]. It would be optimal to assess both: anti-HBs and anti-HBc titers in the study population. However, due to financial constraints, anti-HBc test was arbitrarily chosen to assess the prevalence of HBV infection among HCWs.

Enzyme-linked immune-sorbent assays for the quality detection of anti-HBc total were used (Hoffman-La Roche Ltd., Basel, Switzerland) according to the manufacturer’s guidelines. Each participant donated a blood sample (5 ml) by venipuncture. To ensure confidentiality, a code was given to each subject, for their blood sample and for questionnaire. Laboratory tests were performed at the Pomeranian Medical University Hospital Laboratory in Szczecin. Specimens found to be negative on preliminary screening were considered sero-negative. Blood samples initially tested positive or borderline were re-tested with the use of ELISA to confirm the results. Indeterminate samples were excluded from analysis. Subjects could obtain test results by calling the principal investigator two weeks after the examination. Participants who tested positive for HBsAg were offered an appointment with a specialist.

### Statistical analysis

Data analysis was carried out using STATISTICA PL Version 12.5 (StatSoft Inc., 2016) and the statistical software package R [33]. Frequencies and percentages were used for categorical variables to describe the characteristics of HCWs, and continuous variables were reported as mean ± standard deviation. Anti-HBc prevalence was our primary outcome variable and we aimed to identify explanatory variables significantly associated with this outcome. Bivariate analysis assessed demo-graphic characteristics: age (years), gender, *occupational risk factors*: years in practice, number of working hours per month, job category, type of ward, province (Kuyavia vs. West Pomerania), vaccination against HBV (yes/no), training in infection control (yes/no), SIs in the last 12 months (yes/no), number of SIs (0–1/> 1), glove use (always/rare), SDs use (yes/no); *personal non-occupational risk factors*: ever having blood transfusion (yes/no), having surgery (yes/no), tattooing (yes/no), having multiple sexual partners (yes/no). For categorical variables significance of differences was assessed by chi-square or Fisher exact test; the Mann-Whitney test was used for numeric variables. Significance was presumed when p was < 0.05. For the predicted outcome variable listed above, standard single-outcome logistic regression models were built; all models were reduced by the use of a stepwise selection [34]. Unstandardized regression coefficients in the regression model were used to assess any change in the model. A change in coefficients was compared and used to determine any variable change. For binary data the exponent of the coefficient is interpreted as the odds ratio (OR) [35].

## Results

### Demographic and hospital characteristics

All invited HCWs returned the survey questionnaires and agreed to give a blood sample. Demographic and hospital characteristics of participants are presented in Table 1. Of the total 306 participants, 20.3% worked at hospitals located in the Kuyavia province, the remaining – at hospitals from the West Pomerania. The vast majority of the respondents (88.6%) were females. The mean age of the study population was 47.8 ± 9.0 years (range 23–67 years), the mean length of practice was 25.6 ± 11.0 years (range 0–34 years), about two thirds (63.7%) worked ≤170 h per week. Nurses comprised of 69.9% of participants, followed by midwives (11.8%) and physicians (7.5%). About one fourth of the participants (23.2%) worked at surgical wards and the rest at: internal medicine (21.9%), pediatrics (14.1%), gynecology and obstetrics (13.1%), operating room (11.8%), ICU (8.8%), emergency room (4.6%).

**Table 1:** Demographic characteristics of study participants by province; Poland, 2016–2018 (*n*  =  306)

Characteristic	Total		Kuyavia		West Pomerania		*p*
	*N* 306	% 100.0	*n* 62	% 20.3	*n* 244	% 79.7	
*Gender*							
Male	35	11.4	2	3.2	33	13.5	0.04
Female	271	88.6	60	96.8	211	86.5	0.02
*Age category*							
**≤** 45	109	35.6	18	29.0	91	37.3	0.23
> 45	197	64.4	44	71.0	153	62.7	0.23
*Length of practice*							
**≤** 20	84	27.5	11	17.7	73	29.9	0.06
> 20	222	72.5	51	82.3	171	70.1	0.06
*Number of working hours/week*							
≤ 170	195	63.7	37	59.7	158	64.8	0.55
> 170	111	36.3	25	40.3	86	35.2	0.46
*Job category*							
Physician	23	7.5	1	1.6	22	9.1	0.09
Nurse	214	69.9	55	88.7	159	65.1	< 0.001
Midwife	36	11.8	4	6.5	32	13.2	0.22
Paramedic	17	5.6	2	3.2	15	6.1	0.56
Cleaner	12	3.9	0	0	12	4.9	0.16
No data	4	1.3	0	0	4	1.6	
*Type of ward*							
Surgery	71	23.2	15	24.2	56	23.0	0.80
Gynecology	40	13.1	4	6.5	36	14.8	0.14
Internal medicine	67	21.9	23	37.1	44	18.0	0.02
Pediatrics	43	14.1	7	11.3	36	14.8	0.19
Operating room	36	11.8	5	8.1	31	12.7	0.31
ICU	27	8.8	3	4.8	24	9.8	0.22
Emergency	14	4.6	4	6.5	10	4.1	0.43
No data	8	2.6	1	1.6	7	2.9	
*Personal risk factors*							
Blood transfusion	28	9.2	14	22.6	14	5.7	< 0.001
Surgery in the past	186	60.8	46	74.2	140	57.4	0.02
Tattoo	20	6.5	3	4.8	17	7.0	0.75
Multiple sex partners	105/280	37.5	20	32.3	85	34.8	0.83

### HBV vaccination

**Fig. 1:**
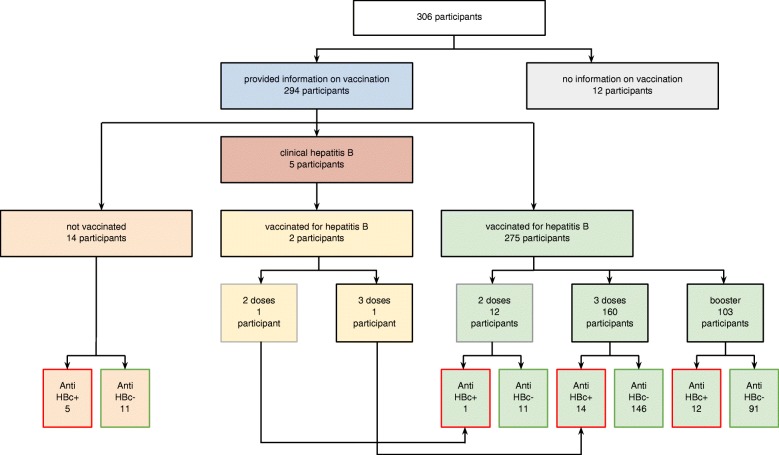
HBV vaccination among study participants in the context of anti-HBc prevalence, Poland, 2016–2018 (*n* = 306)

Figure 1 presents HBV vaccination among study participants in the context of anti-HBc prevalence. Twelve HCWs (3.9%) could not recall if they had received HBV vaccination. Of the remaining 294, 5 (1.7%) had a history of clinical hepatitis B (two of them were vaccinated, one received two doses, the other - a complete course). Fourteen HCWs (4.8%) reported they were not vaccinated, 5 of those were anti-HBc positive. The remaining 275 (93.5%) participants reported HBV vaccination, of these 12 (4.4%) received two doses of vaccine (1 was anti-HBc positive), 160 (58.2%) received the complete course (14 were anti-HBc positive), 103 (37.5%) received the full course plus one booster dose (12 were anti-HBc positive). Of 277 (94.2%) vaccinated HCWs (275 asymptomatic, 2 with a history of clinical hepatitis B), 29 were anti-HBc positive (10.5%). Participants who reported HBV vaccination were then asked about follow-up titers level checked after immunization; only 24.9% reported that they had been checked.

### Risk factors regarding occupationally acquired HBV infection

Risk factors for occupationally acquired HBV infection among hospital staff are presented in Table 2. The vast majority of HCWs were trained in infection control and always used gloves when in contact with potentially infectious body fluids (99.0 and 95.4% respectively). However, over one third of participants (35.9%) reported recapping a needle in the last 12 months. Only 13.7% HCWs reported they did not use SDs at work (30.6% in Kuyavia region, 9.4% in West Pomerania; *p* = 0.02). Among the study participants, 54 did not answer the question about experiencing SIs. Of remaining 252, 27.8% had experienced a SI in the preceding year, with 21.0% experiencing > 1 SI (11.3% in the Kuyavia region, 24.2% in West Pomerania; *p* = 0.03). The vast majority (80.0%) did not report their last SI. The most commonly stated reasons for not reporting an exposure were lack of time and a belief that the patient was not infectious (both 27.6%).

**Table 2:** Risk factors for occupationally acquired HBV infection among hospital staff by region, Poland, 2016–18 (*n* = 306)

Risk factor	Total		Kuyavia		West Pomerania		*p*
	n/N	%	n/N	%	n/N	%	
Not vaccinated against HBV	14/294	4.8	3/61	4.9	11/216	5.1	0.96
Never trained in infection control	3/301	1.0	0/62	0.0	3/244	1.2	0.87
Irregularly used gloves while exposed to blood	14/306	4.6	2/62	3.2	12/244	4.9	0.57
Recapped a needle in the last year	110/306	35.9	21/62	33.9	89/244	36.5	0.70
Did not use safety engineered devices	42/306	13.7	19/62	30.6	23/244	9.4	0.02
Had a history of a sharps injury in the last year	70/252	27.8	12/62	19.4	58/190	30.5	0.09
Had > 1 sharps injuries in the last year	53/252	21.0	7/62	11.3	46/190	24.2	0.03
Did not report the last sharps injury	52/70	80.0	8/12	66.7	44/58	75.9	0.51

### Personal risk factors regarding HBV infection

One in eleventh HCW (9.2%) reported blood transfusion in the past, significantly more in Kuyavia region than in West Pomerania (22.6 and 5.7% respectively; *p* < 0.001). Almost two thirds (60.8%) – had a medical history of surgery, the proportion was significantly higher in the Kuyavia region (74.2 and 57.4% respectively; *p* = 0.02). More than one third of participants (36.5%) reported multiple sexual partners, 6.5% - tattooing; there were no significant differences observed among HCWs from the two studied regions (*p* = 0.83, *p* = 0.75 respectively).

### Seroprevalence of HBV

**Fig. 2:**
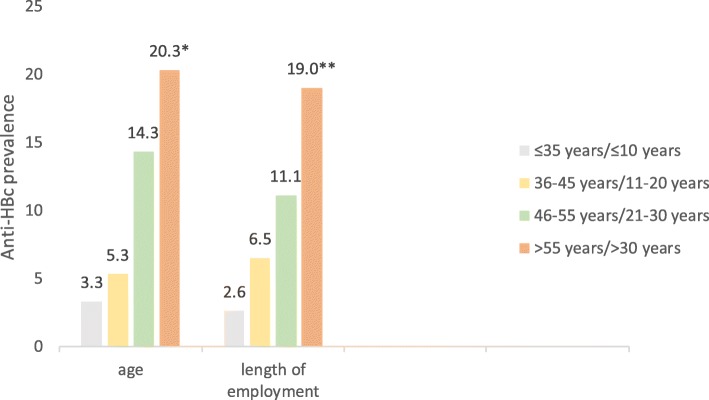
Anti-HBc prevalence among hospital staff by age and length of employment. Poland, 2016–2018, *n* = 306 (chi square for trend: *p < 0.01; **p < 0.01)

From 306 HCWs participating in the study, 37 (12.1%; 95% CI 8.4–15.7%) were positive for anti-HBc; only 11.1% had ever screened themselves for HBV infection. All anti-HBc positive HCWs were referred for HBsAg testing and completed it; none were found to be HBsAg positive. Seropositivity differed significantly in terms of age and length of employment with an evident dose-response effect, i.e. longer exposure, higher prevalence (Fig. 2); chi-square for trend regarding both variables: *p* < 0.01.

The proportion of anti-HBc positive cases was significantly higher in the older age groups (> 45 years) when compared with the younger groups (32/197; 16.2% and 5/109; 4.6%), *p* = 0.005. There was also a significant difference in the proportion of anti-HBc positive cases between HCWs with short (≤20 years) and long (> 20 years) and employment history (4/84; 4.8% and 33/222; 14.9%), *p* = 0.03. Significantly more participants from the Kuyavia hospitals were anti-HBc positive compared to participants from the West Pomeranian hospitals (15/62; 24.2% vs. 22/244; 9.0%); *p* = 0.0003. A significantly higher proportion of anti-HBc positive cases were observed among participants who reported no history of hepatitis B vaccination than those who were vaccinated: 5/14 (35.7%) vs. 32/294 (10.9%); *p =* 0.008.

Midwives (5/36; 13.9%) and nurses (30/214; 14.0%) were professions most having positive anti-HBc screening results followed by physicians 3/23 (13.0%). The highest proportion of anti-HBc positive cases was observed at the emergency (3/14; 21.4%) and internal medicine department (12/67; 17.9%) followed by surgical (11/71; 15.5%) and ICU (4/27; 14.8%) departments; the lowest rate was observed at the operating room (2/36; 5.6%).

No significant difference was observed in the proportion of anti-HBc positive cases between the job categories (*p >* 0.89), hospital departments (*p* > 0.08), gender (*p* = 0.85), and working hours per week (*p* = 0.59). Participants who experienced a SI during the last year of their practice did not differ significantly to get seropositive for anti-HBc compared to those who did not experience (*p* = 0.64); the same refers to the use of SDs (*p* = 0.33) and having surgery/blood transfusion in the past (*p* = 0.81 and *p* = 0.60 respectively) or having a tattoo (*p* = 0.17). A significantly higher proportion of anti-HBc positive cases were observed among HCWs who reported having a single partner than those having multiple sexual partners (30/175, 17.1% vs. 7/105, 6.7%; *p* = 0.02).

Multiple logistic regression analysis showed that the age of HCWs, HBV vaccination status and the province in which participant was working were independent predictors for HBV infection (Table 3). Older participants were more likely to get seropositive for anti-HBc (OR = 4.24, *p* = 0.007) as compared to those of a younger age group. HCWs who were not vaccinated for HBV also more likely to get seropositive (OR = 7.42, *p* = 0.02) compared to those who were vaccinated. Participants from Kuyavia were more likely to get seropositive than those from West-Pomeranian region (OR = 2.69, *p* = 0.03). Interestingly, having a single sexual partner was a predictor of being infected with HBV (OR = 0.30, *p* = 0.04).

**Table 3:** Logistic regression model: association of anti-HBc seropositivity with variables selected with the use of a stepwise approach (OR’s estimates^a^, 95% CIs of OR estimates), Poland, 2016–18; *n*=306

Variable	OR^a^	CI
Age: > 47 years	4.24	1.58–13.32
Vaccination against HBV: no	7.42	1.42–39.49
Working in Kuyavia region: yes	2.69	1.11–6.67
Working hours per week: > 170	1.92	0.80–4.68

^a^Odds ratio = ratio between the two categories tested in each variable, controlling for other variable

## Discussion

### Overview of the results

This is the first multi-center study, conducted among Polish hospital staff from the two provinces with different HBV incidence rates in the general populations, which addresses important aspects of the epidemiology and factors contributing to occupationally acquired HBV infections. Every eighth HCW was anti-HBc seropositive; one in eleven had not been vaccinated or had not received all three doses of the vaccine and three quarters of vaccinated HCWs reported no post-immunization serology. Regarding other occupational risk factors of HBV infection, more than one quarter had experienced a SI, with 80.0% not reporting the incident; only one in nine HCWs had ever performed a screening test for anti-HBc. Recapping needles was reported by more than one third of participants, not using SDs - by 13.7%; the use differed significantly by region. Our study also revealed that the risk of occupationally acquired HBV was heightened by older age, not being vaccinated against HBV and working in the region with a high HBV incidence.

### Anti-HBc seropositivity and HBV vaccination

Prevalence rates of anti-HBc among medical staff vary widely from 1.6% in Mexico [10] to 70% in Albania [6]. The proportion of anti-HBc seropositivity in this study (12.1%) was higher than among Polish surgeons (11.2%), but lower than observed among Polish surgical nurses (16.5%) surveyed in 2009 [12, 27] and among HCWs from the two largest clinical hospitals in the capital – Warsaw (15.7%) surveyed in 2012 [36]. However, it was higher than that reported in our recent study on Polish primary care clinics patients [24].

Although it is possible to assume that infection control procedures - undoubtably vital for all hospital staff - are more important (because of HBV vaccination) in the case of other viral infections, such as HCV and HIV, the results of this study show that Polish HCWs are still at risk of acquiring HBV through their occupations.

Despite universal access to free of charge HBV vaccination, one in twenty participants was not vaccinated; furthermore, 4.7% of vaccinated individuals took only 2 doses of HBV vaccine. Moreover, according to the study results (Fig. 1), 10.5% HCWs vaccinated against HBV presented serological markers of previous or current HBV infection; of all anti-HBc positive participants, 78% had been previously vaccinated. The possible explanation of this finding might be that some HCWs were infected occupationally with HBV in the pre-vaccination era. However, they were not tested for the markers of a previous infection, such as anti-HBs and/or anti-HBc, before vaccination [27]. Due to high prevalence of anti-HBc among previously vaccinated HCWs, which might suggest naturally acquired immunity against HBV, introduction of a test for anti-HBs in a pre-vaccination screening phase for HCWs could be of value. This in turn could reduce the number of HCWs requiring vaccination and avoid unnecessary vaccinations in individuals already being immune [9]. Therefore, such testing should be recommended; HBsAg testing should be performed in seropositive cases.

Additional explanation of this finding could be that some vaccinated individuals - despite feeling well protected - did not seroconvert, especially those who took only two doses of the vaccine, as the response rate is reported as 49–89% in this latter group and decreases with age [37–39]. In patients who completed a 3-dose HBV vaccination the response is typically greater than 95% in young, healthy people, < 90% at age 40 and only 75% at age 60 [40]. As an example, only 83% of patients from primary care clinics, with the mean age of 60 years, surveyed by us previously, gained sero-protection following a 3-dose series [41]. This may also refer to those hospital staff members who were vaccinated against HBV at the older age.

Three quarters of vaccinated HCWs in this study reported no post-immunization serology. As their serological status was not verified one-two months after the vaccination course, the possible lack of immunity remained unnoticed. Meaning that while vulnerable, they could have contracted HBV infection.

Even though HBV vaccination policies are in place in Poland, their implementation still needs improving. According to de Shriver et al., who reported an overview of policies regarding post HBV anti-HBs vaccination testing among HCWs, Poland, together with the Czech Republic and Denmark is in a relatively small group of the European Union countries where there are no strict policies to confirm, monitor or maintain immunity [42]. It should be stressed that testing for anti-HBs after vaccination remains a key preventive tool for those who did not seroconvert because persons who do not respond to an initial 3-dose vaccine series still have a 30–50% chance of responding to a second 3-dose series [19].

Similarly to the results published by Shresta et al. [43], and Nagayo et al. [44], this study showed that HCWs not vaccinated against HBV were much more (OR = 7.42) likely to be anti-HBc positive; this highlights the need for vaccination before initiating a professional career.

### Risk factors for contracting HBV

As stated above, a significant percentage of hospital staff were not aware of their serological status regarding anti-HBs, while some could be non-respondents to the vaccination. Alarmingly, the majority of our study participants did not report the last SI; this is consistent with the pioneer findings of McCormick and Makki from 1980, and other con-current studies which found reporting rates from 3 to 89% [12, 14–17, 45, 46]. The most commonly stated reasons were lack of time and a belief that the patient was not infectious. The latter perception is often misleading; as proven by our previous study on surgical patients from the same hospitals in which the current study was conducted, the vast majority of HBV infected patients were not aware of this fact [22]. The observed, high under-reporting of SIs among study participants impedes detection of individuals without protective anti-HBs levels and the initiation of post-exposure prophylaxis, which in turn puts them at risk of contracting an occupational HBV infection. Of note, acute hepatitis B continues to occur in Poland in unvaccinated cohorts and the prevalence of HBsAg reported in recent studies range from 0.9 to 1.1% [22, 47].

According to European recommendations for the management of HCWs occupationally exposed to HBV, medical staff should be made aware of the clinical and medicolegal relevance of reporting an exposure [48], consider every patient as potentially infectious and recognize the importance of reporting blood exposures in BBI prevention, however, administrative controls at hospital level should also be strengthened to facilitate the reporting process.

Prevalence rates of anti-HBc among medical staff increased with age and length of employ-ment which is in line with other studies [6–11, 27]. A significantly higher (OR = 4.24) rates in older HCWs might suggest the effect of a pre-vaccination era on the incidence of HBV infection. An additional explanation could be that there is a long-lasting exposure of injury events and other blood exposures among hospital staff and therefore the HBV prevalence increases with the age and length of employ-ment of susceptible, non-vaccinated individuals. Nevertheless, it could be concluded that since a younger population of Polish HCWs is immune to HBV infection due to the universal vaccination of newborns and a catch-up vaccination program for teenagers [36, 47], older generation of unvaccinated HCWs constitutes a particular risk group for HBV infection.

There was also a significant difference among the two studied provinces observed in this study, with much higher anti-HBc seropositivity among HCWs from the province with higher HBV incidence in the general population. Moreover, the results of the study showed that HCWs working in the region where the incidence of HBV was higher were about three times (OR = 2.69) more likely to be anti-HBc positive. Although the incidence/prevalence of HBV in the general population might be an important risk factor of an occupationally acquired HBV infection [5], some other contributors could play a role. There was a difference observed between HCWs from the two studied regions regarding job category, with significantly more nurses working at the studied hospitals in the province in with higher anti-HBc seropositivity. As reported in other studies, being a nurse was independently associated with a higher risk of current HBV infection [7]. Several personal factors such as history of blood transfusion and surgery in the past differed among HCWs from the two regions and have been reported in other studies as being associated with an increased risk of HBV infection [7]. Further studies, at a national level should be conducted to better explain the inter-regional differences regarding anti-HBc seropositivity among hospital staff. Nevertheless, constant efforts need to be made in the Kuyavia region to reduce newly acquired HBV infections among hospital staff. As about one third of HCWs did not use SDs, the wider implementation of safety equipment is required to improve healthcare safety.

Our findings indicate that hospital staff are at high risk of SIs and - given the high efficiency of transmission of HBV [5] - are at a considerably high risk of infection. Despite universal access to SDs, 28% of HCWs reported they had experienced a SI in the last year. As SIs are neither fatal nor sufficiently disabling to remove an individual from work, medical staff are at risk of re-injury [17]. And indeed, one in five study participants experienced more than one SI in the 12 months prior to the survey. The rate of SIs observed in our study was similar to that reported in hospital staff in Serbia (25.9%) and Saudi Arabia (31%) [11, 49]. There is a wide variation in the regional or national prevalence of SIs within the past one year among hospital staff worldwide ranging from 19% in Ethiopia to 68% in Uganda [7, 18]. Similarly to the results obtained by Mueller et al. [9], no positive association between SIs and HBV infection was found in this study; this might be due to the fact that in both studies data on SIs were collected from the last 12 months, not the from the respondents whole professional career. It seems that long lasting exposure to SIs rather than self-reported one-year injury statistics should be taken into consideration while assessing the risk of acquiring an occupational BBI. Some other authors found [11, 50, 51] SIs sustained during their professional career to be significantly associated with risk of HBV infection.

Of note, the percentage of HCWs experiencing multiple sharps-related injuries (> 1 SI) differed across provinces, with West Pomerania reporting a two times higher percentage than Kuyavia. This information is important for intervention development, as it provides an added dimension to the scope of the existing problem within each region. Although educational interventions - such as training in infection control with an emphasis on SI prevention - should cover all HCWs, hospitals representing the West Pomeranian province need special attention and support.

The highest proportion of anti-HBc positive HCWs was observed at an emergency and internal medicine department. This finding is in line with the study of Keller et al. who reported a high pre-valence of HBV infection in a population of the US patients in an emergency room [23], and Wicker et al. [14] who found that the nominal highest risk of viral infection was carried by HCWs in the field of internal medicine because of the higher prevalence of BBIs in their patients, as well as a high rate of SIs.

After controlling for other variables, having a single sexual partner remained significantly associated with a higher risk of HBV infection. This finding is at variance with what has been reported in other studies where the risk of sexual transmission of HBV was elevated in unvaccinated hetero-sexual persons with multiple sexual partners [52, 53]. One possible explanation could be a prolonged exposure to unprotected sex with a regular partner, asymptomatically infected with HBV, in contrast to protected sexual contacts with irregular partners. Transmission of HBV has been found to correlate with duration of sexual activity [54]. A more precise query relating to having unprotected sexual intercourse with an unknown partner(s), rather than a query about sexual contacts with multiple sexual partners, could better assess the possible association between the risk of sexual transmission of HBV. Further studies are needed to better assess this issue.

### Limitations

Potential limitations did exist in this study. The primary limitation was that only samples from HCWs present at the ward the day the study was conducted were tested, which would likely result in an over- or underestimation of the proportion of HBV infections among medical staff. Secondly, recollecting information on risk factors could introduce recall bias [55, 56]. Thirdly, infection control practices were based on the respondents reported response, not on the direct observation of their practices which could have resulted in an over-estimation of compliance. The authors arbitrarily restricted sharps injury reporting to “within the last year”. Further studies assessing the number of SIs sustained by a HCWs during their professional career and within the interval between the imple-mentation of the directive and survey administration would be of value. The social desirability of preventive health behaviors may lead participants to over-report vaccination. Although the rates for HBV infections may be representative for the two provinces selected for the purpose of this study, variation can be also expected. Finally, the cross sectional study design did not allow for the assessment of temporal associations [55].

## Conclusions

A relatively high seroprevalence of anti-HBc was found in Polish hospital staff, especially those working at the emergency and internal medicine departments, with the older generation particularly constituting a risk group. The study provides evidence of the protective effect of HBV vaccine, highlighting the need for universal vaccination of all HCWs before they start their professional career. Protection of HCWs against HBV among hospital staff was below the desired level: one in eleven employees had not been vaccinated or had not received all three doses of the vaccine; three quarters of vaccinated HCWs reported no post-immunization serology. Of note, the high prevalence of anti-HBc (10.5%) among previously vaccinated HCWs might suggest naturally acquired immunity against HBV [9]. Therefore, anti-HBs testing should be recommended for hospital staff before immunization against HBV to avoid unwarranted vaccinations.

The results of this and other studies show there is a complex assortment of risks of exposure to HBV and it is difficult to point out the biggest contributors to occupationally acquired HBV infections in healthcare sector [7–9]. However, the findings raise concerns related to compliance with infection-control procedures, so transparently expressed in the EU Council Directive from 2010 [25], such as safe work practices, HBV post-immunization serology and reporting of exposures. Although most HCWs in this study attended training in infection control, so far, it seems that such training is not effective in Polish hospitals, as it does not influence compliance. Therefore, training methods should be modified and intensified - with particular emphasis to SIs risk factors and post-exposure reporting procedures - to increase awareness and tighter infection-control measures.

In conclusion, complete HBV vaccination coverage, followed by strict policies to confirm immunity, better compliance with infection-control practices and widespread implementation of SDs should be enforced in Polish hospitals to protect hospital staff from occupationally acquired infections. A continued increase in the use of safety devices at Polish hospitals and universal HBV vaccination of a younger population of HCWs due to population-based programs [36, 47] offer hope to limit the number of new infections among hospital staff.

## Additional file

Additional file 1: Seroprevalence of anti-HBc, risk factors of occupationally acquired HBV infection and HBV vaccination among hospital staff in Poland: A multicenter study. An English language version of the study questionnaire. (DOCX 40 kb)

## Abbreviations

95%-CI: 95%-confidence interval
anti-HBc: Hepatitis B core antibody
ELISA: Enzyme-linked immunosorbent assay
EU: European Union
HBe Ag: Envelope antigen of the hepatitis B virus
HBs Ag: Surface antigen of the hepatitis B virus
HBV: Hepatitis B virus
HCW: Health care worker
OR: Odds ratio
SED: Safety engineered device
SI: Sharps injury
