# Supplementary material for: Seroprevalence of anti-HBc, risk factors of occupationally acquired HBV infection and HBV vaccination among hospital staff in Poland: a multicenter study
Source: BMC Public Health. 2019 Mar 12;19:298. doi: 10.1186/s12889-019-6628-1 (PMC6417128; doi:10.1186/s12889-019-6628-1)
Supplement: Supplementary file 1 — Seroprevalence of anti-HBc, risk factors of occupationally acquired HBV infection and HBV vaccination among hospital staff in Poland: A multicenter study. An English language version of the study questionnaire. (DOCX 40 kb) [file 12889_2019_6628_MOESM1_ESM.docx]

**Risk factors of occupationally acquired HBV infection**

**among hospital staff in Poland**

*Code:  Date………………………

1. **Demographic characteristics**
2. **Age (years) **
3. **Gender**

- M 1
- F 2

1. **Length of practice (years) **
2. **Hospital ward (type), please specify ……………………………**
3. **Type of hospital**

- Urban 1
- Teaching 2
- Provincial 3
- Other type 4

6. **Job category**

- Physician 1
- Nurse 2
- Paramedic 3
- Cleaner 4
- Midwife 5

1. **Province**

- Kuyavia 1
- West Pomerania 2

**__________________________________________________________________________________**

1. **Selected occupational risk factors for contracting HBV infection**

**8. How many hour do you work per week?**

- up to 85 1
- 86-170 2
- >170 3

1. **What is your serological status regarding HBV infection?**

- Clinical hepatitis B 1
- Vaccination: full course 2
- Vaccination: 1 or 2 doses 3
- Vaccination: full course & booster 4
- Unvaccinated 5
- Don’t remember 6

**10. Was follow-up titers level checked after HBV immunization?**

- Yes 1
- No 2
- Don’t remember 3

**11. Have you attended training in infection control?**

- Yes 1
- No 2
- Don’t remember 3

12. Are safety engineered devices (SDs) used in your ward?

- Yes 1
- No 2

**13.** **Did you recap a needle in the last year?**

- Yes 1
- No 2
- Don’t remember 3

**14.** **Did you sustain any sharps injuries in the last year?**

- Yes (how many?) 
- No 2
- Don’t remember 3

**15**. **Was the last sharps injury reported to the hospital infection control unit?**

- Yes 1
- No 2
- Don’t remember 3

**16. If not, please state the reason for not reporting the exposure**

- Did not know how to report exposure 1
- Source patient was thought not to be infectious 2
- Insufficient time to report 3
- Reporting would not result in avoiding infection 4
- Other reasons ………………………………………………………… 5

**17. Have you ever been tested for HBV infection?**

- Yes 1
- No 2
- Don’t remember 3

**18**. **Do you use gloves while performing procedures exposing to blood contacts?**

- Always 1
- Rare 2
- Never 3

19. If “rare” or “never”, please state the reason for the irregular use

- Gloves were not available 1
- Source patient was thought not to be infectious 2
- Insufficient time 3
- Gloves interfered with providing good patient care 4
- Other reasons…………………………… 5

1. **Selected non-occupational risk factors for contracting HBV infection**

**20. Have you had surgery in the past?**

- Yes 1
- No 2
- Don’t remember 3

**21. Have you had a blood transfusion?**

- Yes 1
- No 2
- Don’t remember 3

**22. Have you had multiple sexual partners?**

- Yes 1
- No 2
- Don’t remember 3

**23. Do you have any tattoos?**

- Yes 1
- No 2
- Don’t remember 3

**THANK YOU FOR COMPLETING THE QUESTIONNAIRE**
